# Supplementary material for: Role of Lung Ultrasound in the Management of Patients with Suspected SARS-CoV-2 Infection in the Emergency Department
Source: J Clin Med. 2022 Apr 7;11(8):2067. doi: 10.3390/jcm11082067 (PMC9025104; doi:10.3390/jcm11082067)
Supplement: Supplementary file 1 [file jcm-11-02067-s001.zip › table S2 modificata.pdf]

	All Patients (1826)	COVID-19 + (617)	COVID-19 - (843)	P value
Female sex	957 (52,4%)	301 (48,8%)	453 (53,7%)	0,089
Hypertension	561 (30,7%)	238 (38,6)	261 (31%)	0,074
Diabetes	171 (9,4%)	69 (11,2%)	77 (9,1%)	0,168
COPD	165 (9,0%)	66 (10,7%)	89 (10,5%)	0,198
Asthma	70 (3,8%)	18 (2,9%)	42 (5%)	0,200
Other lung disease	67 (3,7%)	18 (2,9%)	40 (4,7%)	0,242
Ischaemic cardiac disease	148 (8,1%)	49 (7,9%)	85 (10,1%)	0,002
Cancer active	101 (5,5%)	25 (4,1%)	65 (7,7%)	0,003
CKD	122 (6,7%)	48 (7,8%)	62 (7,4%)	0,068
Stroke	90 (4,9%)	36 (5,8%)	45 (5,3%)	0,204
Immune deficency	28 (1,5%)	7 (1,1%)	12 (1,4%)	0,293

Table S2. Comorbidities of study patients; CKD: chronic kidney disease; COPD: chronic obstructive pulmonary disease. Categorical variables are presented as frequencies and percentages.
