# Supplementary material for: Role of Lung Ultrasound in the Management of Patients with Suspected SARS-CoV-2 Infection in the Emergency Department
Source: J Clin Med. 2022 Apr 7;11(8):2067. doi: 10.3390/jcm11082067 (PMC9025104; doi:10.3390/jcm11082067)
Supplement: Supplementary file 1 [file jcm-11-02067-s001.zip › Table S3.pdf]

Symptoms	All Patients (1826)	COVID-19 + (617)	COVID-19 - (843)	P value
Fever	1325 (72,6%)	498 (80,7%)	567 (67,3%)	0,000
Dyspnea	641 (35,1%)	243 (39,4%)	283 (33,6%)	0,305
Cough	803 (44,0%)	305 (49,4%)	324 (38,4%)	0,000
Conjunctivitis	42 (2,3%)	8 (1,3%)	27 (3,2%)	0,163
Pharyngodynia	203 (11,1%)	38 (6,2%)	107 (12,7%)	0,000
Headache	225 (12,3%)	55 (8,9%)	99 (11,7%)	0,000
Asthenia	317 (17,4%)	112 (18,2%)	141 (16,7%)	0,477
Myalgia/arthralgia	219 (12,0%)	76 (12,3%)	98 (11,6%)	0,900
Diarrhea	300 (16,4%)	86 (13,9%)	146 (17,3%)	0,071
Anosmia	73 (4,0%)	30 (4,9%)	25 (3,0%)	0,283
Ageusia	146 (8,0%)	56 (9,1%)	53 (6,3%)	0,033
Chest pain	98 (5,4%)	16 (2,6%)	51 (6,0%)	0,016

Table S3. Main symptoms reported by patients on presentation to the emergency department; categorical variables are presented as frequencies and percentages.
