# Supplementary material for: Role of Lung Ultrasound in the Management of Patients with Suspected SARS-CoV-2 Infection in the Emergency Department
Source: J Clin Med. 2022 Apr 7;11(8):2067. doi: 10.3390/jcm11082067 (PMC9025104; doi:10.3390/jcm11082067)
Supplement: Supplementary file 1 [file jcm-11-02067-s001.zip › table S4.pdf]

	All patients (1826)	COVID-19 + (617)	COVID-19 - (843)	P value
Age (years)	57,0 ± 21,2	62,3 ± 19,3	57,3 ± 21,7	0,000
SBP (mmHg)	128,3 ± 21,0	125,9 ± 20,2	129,4 ± 22,2	0,032
DBP (mmHg)	75,5 ± 12,8	74,6 ± 12,4	75,6 ± 13,4	0,159
MAP (mmHg)	68,4 ± 42,4	76,7 ± 36,1	68,8 ± 43,2	0,118
CR (bpm)	88,2 ± 16,9	88,9 ± 16,7	89,0 ± 17,1	0,95
RR (a/min)	18,8 ± 5,2	19,7 ± 5,5	18,7 ± 5,2	0,000
Temperature (°C)	36,9 ± 0,7	37,1 ± 0,8	36,9±0,7	0,000

Table S4. Age and vital signs of patients on emergency department admission; SBP, systolic blood pressure; DBP, dyastolic blood pressure; MAP, mean arterial pressure; CR, cardiac rate; RR, respiratory rate. Continuous variables are expressed as mean ± standard deviation.
