# Supplementary material for: Role of Lung Ultrasound in the Management of Patients with Suspected SARS-CoV-2 Infection in the Emergency Department
Source: J Clin Med. 2022 Apr 7;11(8):2067. doi: 10.3390/jcm11082067 (PMC9025104; doi:10.3390/jcm11082067)
Supplement: Supplementary file 1 [file jcm-11-02067-s001.zip › table S9.pdf]

	AUC	STD ERROR	Asymptotic sig.	IC 95%
**LUS score**	0,816	0,120	0,010	0,581 – 1,000
**P/F**	0,054	0,032	0,000	0,000 – 0,117
**DELTA (A-a)**	0,825	0,127	0,008	0,575 – 1,000
**DELTA increase**	0,777	0,081	0,023	0,619 – 0,936

Table S9. Data derived by ROC analysis for 30-days mortality in COVID-19 patients; AUC, area under the curve; IC, confidence interval.
