# Supplementary figures and images for: Role of Lung Ultrasound in the Management of Patients with Suspected SARS-CoV-2 Infection in the Emergency Department
Source: J Clin Med. 2022 Apr 7;11(8):2067. doi: 10.3390/jcm11082067 (PMC9025104; doi:10.3390/jcm11082067)

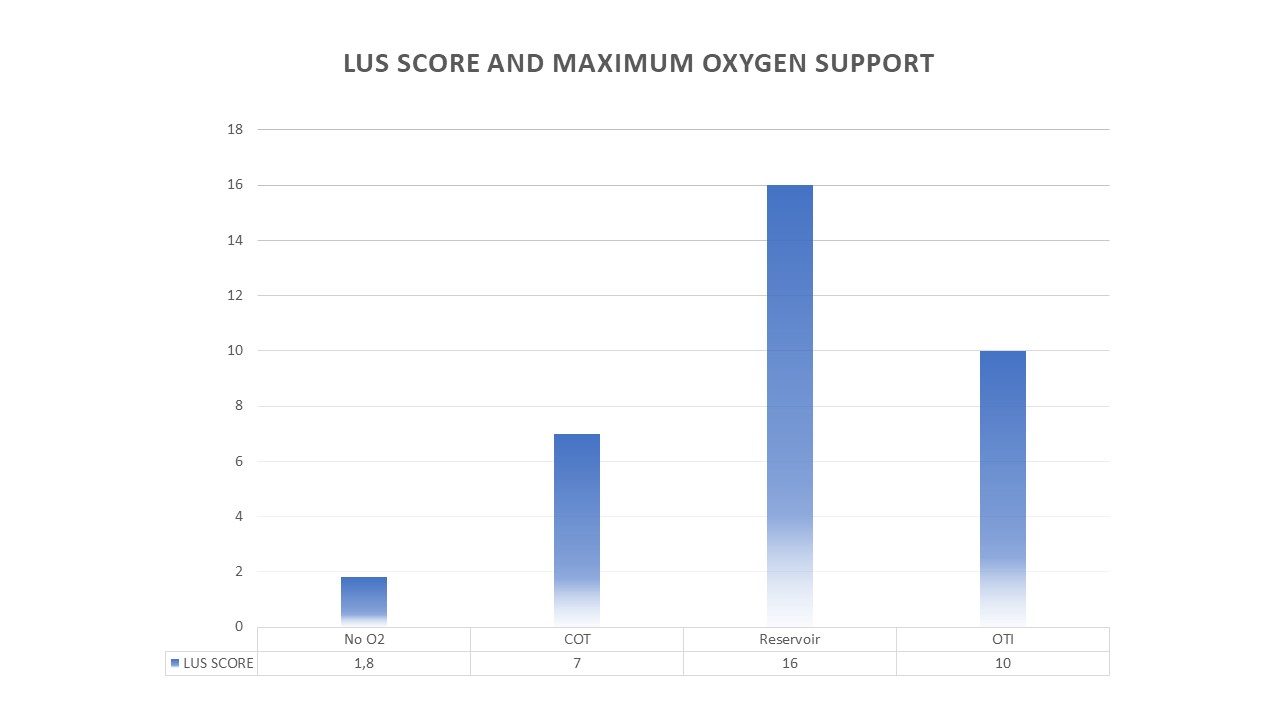

Supplement: Supplementary file 1 [file jcm-11-02067-s001.zip › suppl mat 1- figure S1.jpg]

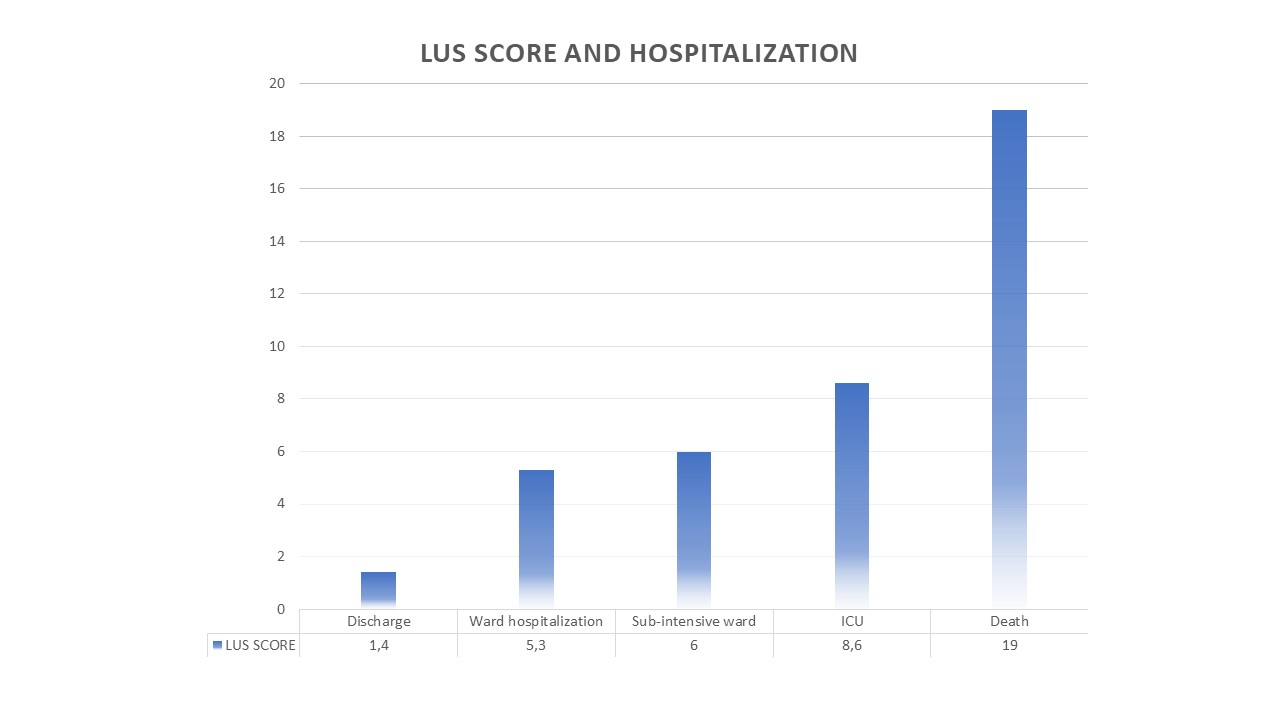

Supplement: Supplementary file 1 [file jcm-11-02067-s001.zip › suppl mat 2- figure S2.jpg]

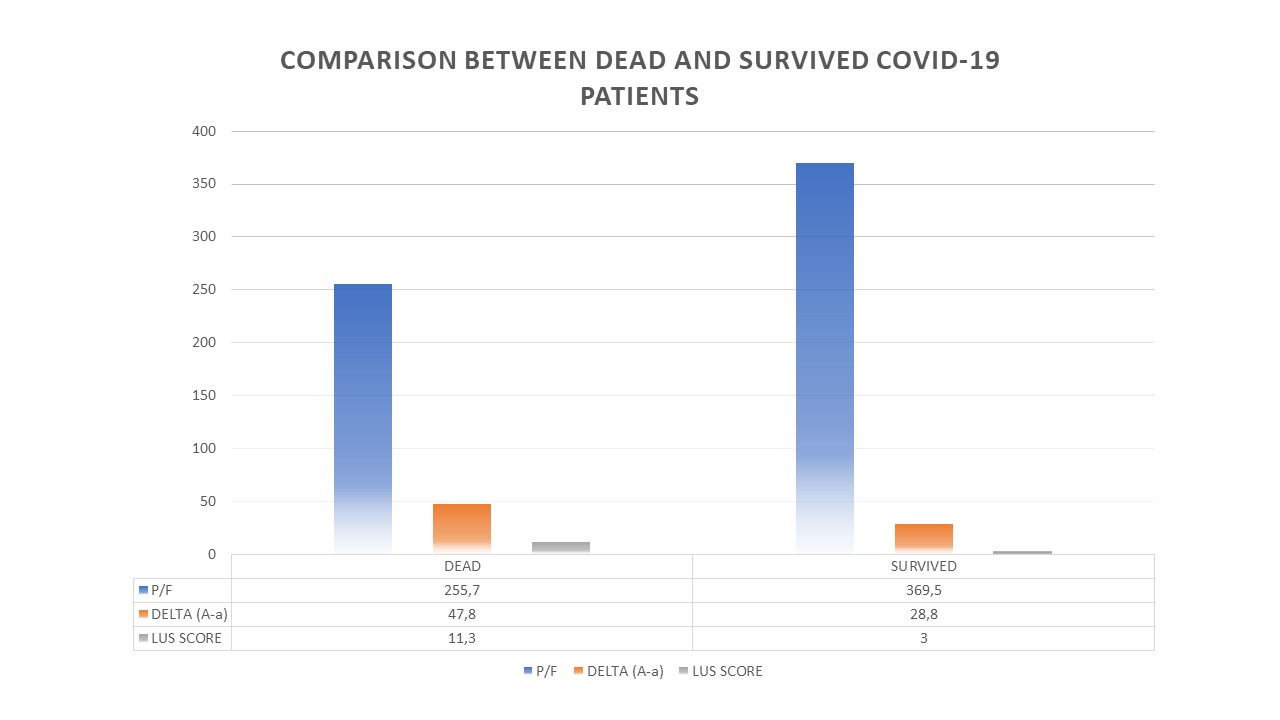

Supplement: Supplementary file 1 [file jcm-11-02067-s001.zip › supple mat 3- figure S3.jpg]
